# Nitric oxide signaling in plants

**DOI:** 10.3389/fpls.2013.00553

**Published:** 2014-01-16

**Authors:** Emmanuel Baudouin, John T. Hancock

**Affiliations:** ^1^Plant Cellular and Molecular Physiology, Sorbonne Universités, UPMC Univ Paris 06, UR5Paris, France; ^2^Plant Cellular and Molecular Physiology, CNRS, EAC 7180Paris, France; ^3^Department of Health and Life Sciences, Centre for Research in Plant Science, Genomics Research Institute, University of West of EnglandBristol, UK

**Keywords:** defense responses, hormones, nitric oxide, post-translational modifications, ROS, signaling

Nitric oxide (NO) is now seen as a vitally important molecule in many biological systems. Once it was identified in mammalian systems in 1987 (Palmer et al., 1987) it was only a matter of time before researchers hunted for its presence in plants, with the first such reports being published in 1998 (Delledonne et al., 1998; Durner et al., 1998). Now 15 years later the interest in NO and its roles in plants is as eagerly investigated as ever, with an ever increasing number of papers published in the area each year. However, the field is not short of controversies, and it would certainly be fair to say that there is still much to be learnt in the area of NO biology. Here, a collection of papers by the many of the most active research groups in the field has been brought together. Some have contributed original research, others have written reviews to enable readers to get a up-to-date view of specialist areas in the field, while others still have written opinion articles, which give their views of the state-of-play in NO research as they see it.

One of the controversies which has caused problems over many years is the way in which NO should be measured in plants. Gupta and Igamberdiev (2013) have contributed an opinion paper and propose that at least two different methods should be used to be sure that NO is truly being measured. This is sound advice and hopefully a strategy that will be adopted by many in the field in the future. D'Alessandro et al. (2013) continue this theme of caution with a paper on the use of cPTIO. This compound is often employed as a scavenger to confirm that NO is being detected, but it is also used as a means to measure the presence of NO when coupled to electron paramagnetic resonance (EPR). These authors report a systematic investigation into the scavenging of cPTIO and discuss the reliability of such use and as an EPR probe.

With the above caveats in place, there seems to be little doubt that NO is present in plants, but a second controversy surrounds the precise sources for its generation. In mammalian systems there are well-characterized nitric oxide synthase (NOS) enzymes but their existence in plants has been thrown into doubt (Hancock, 2012). Correa-Aragunde et al. (2013) compare and discuss the structures of the different NOS enzymes structures across prokaryotes and eukaryotes. In particular they emphasize the presence of such an enzyme in the unicellular microalgae *Ostreococcus tauri*. They do not rule out that higher plants may too have a form of NOS, but only time will tell if they are correct in this opinion. Mur et al. (2013) take a more holistic view of NO metabolism in plants and consider the balance of NO generation, exposure of plants to NO from external sources and the scavenging activity for NO within the plant tissue. They further discuss the impact of the exposure of plant tissues to NOx (NO and NO_2_) derived from microbial activity, and ask the question as to whether there is an impact from nitrate metabolism on the overall accumulation of NO in plants.

Since 1998 many studies have shed light on the profound effects NO exerts on plant cell functioning and whole plant development and response to environmental cues. As a first example, Arc et al. (2013) discuss the role of NO in the breaking of seed dormancy and germination. This review presents aspects of NO chemistry in seeds and concludes that NO-dependent protein modification is important during seed germination. Protein modification by NO is a key mechanism when considering downstream effects and is further discussed by others, as described below. As a second example Boscari et al. (2013) address the role of NO in root nodules and in this mini-review question whether NO is actually used as an intermediate in N_2_ fixation. Silva and Carvalho (2013) also consider the impact of NO on root nodules, but here the role of glutamine synthetase is the focus, with the suggestion that this enzyme is involved in NO signaling responses in this context. At the other end of the plant, it is also well documented that NO has a pivotal function in the control of stomatal apertures, and this is discussed in a review article by Gayatri et al. (2013), with a particular focus on the interplay between NO and cytosolic pH, reactive oxygen species (ROS) and free calcium ions. The different reviews open routes for future developments in their particular field but all underline that integrating NO into the global redox network is of topical importance. This aspect is specifically discussed in two reviews presented below.

One of the original roles that were determined for NO in plants was in plant defence. In an original article Schlicht and Kombrink (2013) investigate NO function in the defence against fungi. They report the accumulation of NO at infection sites, and suggest that there is a correlation between resistance phenotypes and NO production, both in its timing and accumulation. Groß et al. (2013) further tackle the topic of plant defence by reviewing the interaction of NO with antioxidants and prooxidants. They discuss the reaction of NO with ROS and the formation of other reactive compounds such as peroxinitrite, as well as the removal of NO through the action of non-symbiotic hemoglobins (nsHb). Wang et al. (2013) also consider the role of the interaction of NO and ROS in a review which focuses on the cross-talk between these two important signaling pathways. Their discussion considers plant defence through the role of NO and ROS on the hypersensitive response, but also on leaf senescence and other types of programmed cell death (PCD).

The intimate relationship between NO and antioxidants is particularly illustrated with the case of glutathione, one of the major antioxidants in cells that is hugely important in the control of ROS metabolism. Indeed, it is well established that there is a key reaction between glutathione and NO which results in the formation of S-nitrosoglutathione (GSNO)—this is the discussion point of an opinion article by Corpas et al. (2013). The authors summarize GSNO metabolism and suggest that investigating GSNO occurrence and function in plant cells, along with other NO reaction products, will be an important issue for the future understanding of the role of NO in plant development and stress responses. Illustrating this opinion the enzymes S-nitrosoglutathione reductases (GSNORs) are the subject of an original article by Xu et al. (2013), where the importance of these enzymes is discussed in view of their role in development and defence. They use bioinformatics and structural modeling to show the location of GSNORs and identify conserved amino acids, which are vital to their role.

The mechanisms that allow NO perception and its conversion into physiological responses have been paid much attention in the last years. In that view proteomics has been used successfully to determine the proteins that may react directly with signaling compounds including NO. Romero-Puertas et al. (2013) consider the formation of the main protein modification that is S-nitrosylation, during abiotic stress. The reaction of NO with protein thiols is an immensely important way in which NO may have its effects in all biological systems, with plants being no exception. París et al. (2013) continue this discussion in a mini review by considering how S-nitrosylation is involved in the workings of hormone networks. Future routes for investigating S-nitrosylation function in plant cells are proposed. Mengel et al. (2013) focus on the role of S-nitrosylation in a particular cell compartment, i.e., the nucleus, and review the effects of NO on gene transcription, with comparison to the work that has been carried out on animal systems. On the other hand, Sehrawat et al. (2013) suggest that the depletion of RuBisCo from samples would be an advantage to broaden the S-nitrosylated protein atlas, and they report on new cold-responsive S-nitrosylated targets in *Brassica juncea*. Another NO-based protein modification is tyrosine nitration and Blume et al. (2013) report the regulation of cytoskeleton organization via the tyrosine nitration of α-tubulin.

How NO, or indeed any other compounds, are able to bring about control of cell function means that there must be an influence on cell signaling events. As can be seen from above NO has an impact on other signaling such as that carried out ROS—as discussed by Wang et al. (2013) for example—as well as having effects on signaling proteins by S-nitrosylation or tyrosine nitration. But other signaling pathways and components can be affected too. Guillas et al. (2013) discuss its impact on sphingolipids, and again use comparisons with the work in animals to aid in the understanding of the way such molecules interact in plants. They conclude that although the generic idea may be common across eukaryotes the details will be different. On the other hand in a mini review Salmi et al. (2013) consider how NO may mediate the signaling by extracellular nucleotides. Such extracellular signals have been found to induce NO generation in plant cells, and this paper discusses this with particular reference to auxin signaling and plant growth. This underlines the crucial role of NO in the control of events initiated by phytohormones that is the subject of a review by Freschi (2013). Here the focus is on the pathways that regulate metabolism and development but this paper also brings together many elements of NO signaling discussed by others in this collection, including gene expression, defence responses, and post-translational protein modifications.

The field of NO generation and effects in plants has moved a long way since it was first suggested in 1998, and this collection of papers discusses many aspects of the area as it stands today. However, it also highlights that there is still a long way to go before there is a clear understanding of how NO is made by plants cells and how NO fits into the signaling that controls so many key aspects of plant growth and development. However, several of the authors have given their opinions and ideas that will be useful to steer the direction of plant NO research in the future.
